# An optimized protocol for evaluating pathogenicity of *VHL* germline variants in patients suspected with von Hippel-Lindau syndrome: Using somatic genome to inform the role of germline variants

**DOI:** 10.1016/j.mex.2022.101761

**Published:** 2022-06-18

**Authors:** Diane R. Koeller, Danielle K. Manning, Alison Schwartz, Anu Chittenden, Connor P. Hayes, Feruza Abraamyan, Huma Q. Rana, Neal I. Lindeman, Judy E. Garber, Arezou A. Ghazani

**Affiliations:** a Division of Cancer Genetics and Prevention, Dana-Farber Cancer Institute, Boston, Massachusetts, USA; b Department of Pathology, Brigham and Women's Hospital, Boston, Massachusetts, USA; c Division of Genetics, Department of Medicine, Brigham and Women's Hospital, Boston, Massachusetts, USA; d Division of Population Sciences, Dana-Farber Cancer Institute, Boston, Massachusetts, USA; e Harvard Medical School, Boston, Massachusetts, USA

**Keywords:** LOH, Loss of heterozygosity, von Hippel-Lindau syndrome, VHL syndrome, Integrated somatic and germline NGS, Germline variant interpretation, Loss of heterozygosity (LOH), Von Hippel-Lindau syndrome, VHL gene

## Abstract

The interpretation of hereditary genetic sequencing variants is often limited due to the absence of functional data and other key evidence to assess the role of variants in disease. Cancer genetics is unique, as two sets of genomic information are often available from a cancer patient: somatic and germline. Despite the progress made in the integrated analysis of somatic and germline findings, the assessment of pathogenicity of germline variants in high penetrance genes remains grossly underutilized. Indeed, standard ACMG/AMP guidelines for interpreting germline sequence variants do not address the evidence derived from tumor data in cancer. Previously, we have demonstrated the utility of somatic tumor data as supporting evidence to elucidate the role of germline variants in patients suspected with VHL syndrome and other cancers. We have leveraged the key elements of cancer genetics in these cases: genes with expected high disease penetrance and those with a known biallelic mechanism of tumorigenicity. Here we provide our optimized protocol for evaluating the pathogenicity of germline *VHL* variants using informative somatic profiling data. This protocol provides details of case selection, assessment of personal and family evidence, somatic tumor profiles, and loss of heterozygosity (LOH) as supporting evidence for the re-evaluation of germline variants.

## Graphical abstract

**Figure:**
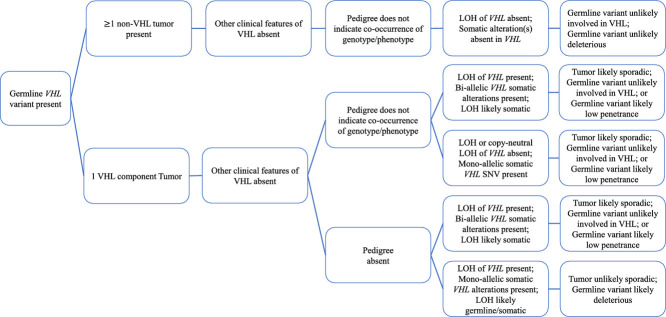

Specifications table

**Table:** 

Subject Area:	Biochemistry, Genetics and Molecular Biology
More specific subject area:	Integrated interpretation of somatic and germline genomic data in cancer
Protocol name:	Evaluation of pathogenicity of germline *VHL* variants in patients suspected with VHL syndrome using somatic signature profile as supporting evidence
Tools/method:	N/A
Experimental design:	A retrospective chart review was performed to identify informative candidates for this study. Germline gene panel testing, somatic tumor profiling, LOH assessment, personal medical and family history collection were performed for all subjects.
Trial registration:	N/A
Ethics:	Appropriate institutional informed written consent was obtained prior to testing and data sharing
Value of the Protocol:	• Current protocols for the interpretation of germline genetic variants do not address the contribution of somatic data in cancer. This protocol customizes the evaluation of germline *VHL* variants in disease using informative somatic tumor data. • The refined interpretation of germline *VHL* variants and phenotypical implications can aid the management of patients with suspected VHL syndrome. • This protocol can be extended to other cancers with expected high disease penetrance and bi-allelic loss of function etiology.

## Introduction

The current ACMG/AMP guidelines [1] for the evaluation of pathogenicity of germline sequence variants do not account for the somatically derived information from patients’ tumor. While somatic data may not be informative in elucidating the role of germline variants in all cancer types, they can play an invaluable role in cancers with an expected high penetrance etiology and those with a biallelic mechanism of tumorigenicity. The integrated somatic and germline analysis has been reported beneficial in personalized therapeutic practice [2], tumorigenesis and cancer progression [3], inference on germline allele penetrance [4], [5], [6], [7], and gene discovery [8]. However, a systematic application of integrated somatic and germline data has not been presented in the assessment of germline variant pathogenicity.

We have previously demonstrated the value of tumor-derived information in the assessment of germline variants in patients who do not meet the classic criteria for clinical cancer diagnosis [4], [5], [6]. In these cases, the presence of the following key factors was proven helpful: First, genes for which biallelic inactivation through loss of heterozygosity (LOH) is a known mechanism of inactivation and disease. This allows the evaluation of germline and somatic contributions to LOH. Second, genes and alleles that are expected to be highly penetrant for a known cancer condition. The rationale is if high penetrant alleles are present, the disease phenotype is expected to be expressed. The von Hippel-Lindau disease (VHL) syndrome fits these criteria. Biallelic inactivation of *VH*L gene is a known and expected mechanism of *VHL* loss of function [9]. Moreover, the *VHL* gene is associated with an expected high lifetime risk of VHL multiple component tumors in individuals harboring inherited deleterious variants. Individuals with VHL disease are estimated to have a 90% chance of developing the disease by age 60 years [5,^10^,11]. Component tumors include renal cell carcinomas, hemangioblastomas, renal cyst, pancreatic cysts, pheochromocytoma, neuroendocrine tumors, endolymphatic sac tumors, and epididymal and broad ligament cysts [5,^12^,13].

With this background, we have utilized somatic genetic data to optimize the assessment of germline *VHL* variants in patients who do not exhibit the classic syndromic phenotype of VHL disease. Here, we provide a step-by-step procedural detail and the rationale for each step that enables the re-evaluation of germline *VHL* variants and their potential functional contribution to disease. This protocol may ultimately improve the clinical management of suspected VHL patients, and the concept may be further extended to other cancer genetic syndromes with similar characteristics.

## Description of protocol

In the ACMG-based assessment of germline variants, often strong evidence in support of pathogenicity, such as functional studies and/or increased disease prevalence, are not present in the literature. In cancer, the assessment of these germline variants can be aided by robust evidence from tumor genetics, mechanism of tumorigenicity, and the somatic genetic signature. By leveraging tumor derived data, we have created a customized protocol for the assessment of germline *VHL* variants in patients positive for *VHL* germline variants and suspected of having the VHL disease.

## Design

**Fig. 1:**
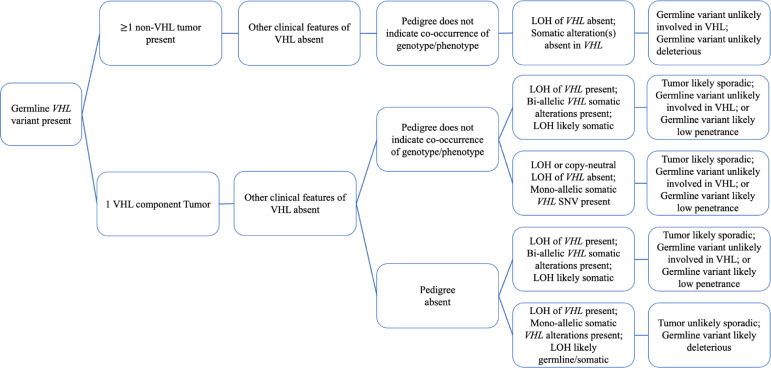
An optimized protocol for the evaluation of pathogenicity of germline *VHL* variants in patients suspected with VHL syndrome using somatic signature profile as supporting evidence. Patients positive for a germline *VHL* variant with a phenotype consistent with >1 VHL component tumors were evaluated for the classic VHL disease and therefore excluded from this assessment. Patients included in this assessment are those positive for a germline *VHL* variant with tumor(s) not consistent with VHL syndrome, or those with only one component tumor of VHL syndrome. A systematic evaluation of personal and family history of patients along with tumor derived somatic data and the state of LOH of *VHL* alleles provided supporting evidence in functional classification of *VHL* germline variants.

A retrospective chart review was performed to identify informative candidates for this study. Personal and family history of patients positive for *VHL* germline variants were evaluated. Pathology and somatic data were assessed for the type of tumor and the presence of LOH in relevant tumors. After comprehensive assessment, germline *VHL* variants were re-evaluated. The diagram of the protocol's decision tree and processes is illustrated in Figure 1.

## Patient Selection

The study cohort consisted of patients seen at Dana-Farber Cancer Institute enrolled in the PROACTIVE research program [4], [5], [6]. Patients with a personal history of cancer with known germline *VHL* variants with non-syndromic VHL phenotypes and/or phenotypes discordant with genotype were selected for germline evaluation (Figure 1). Syndromic VHL was defined as the presence of more than one VHL component tumors and/or multiple different tumors or features.

### Exclusion criteria for probands

The presence of more than one component tumor associated with VHL syndrome in the proband or their family was used as an exclusion criterion. The rationale for this exclusion is that patients in this category will likely meet the classic VHL evaluation and/or diagnosis requirements.

### Inclusion criteria for probands

Inclusion requirements were the presence of positive germline *VHL* variant test results and the presence of tumor testing, pathology results and somatic profile. Both germline and somatic results were required for the integrated genomic evaluation.

## Personal medical history

A detailed medical history was collected focusing on oncologic history, primary and metastatic tumors, pathology records, and any available imaging. The type of tumors in each patient was assessed. Based on tumor types, patients were categorized into two groups (Figure 1): patients with tumor(s) not consistent with what is expected for VHL syndrome (e.g., bladder cancer), and patients with only one component tumor of VHL syndrome that do not meet the criteria of classic VHL diagnosis (e.g., clear cell renal cell carcinoma).

## Family history

The probands' pedigrees, were evaluated, focusing on cascade testing, and oncologic history. The pedigree members with confirmed germline *VHL* variants were evaluated for features of VHL syndrome. The pedigrees were also evaluated to assess whether there was any associated presence of the *VHL* germline variant with component tumors. These steps include:

### Cascade testing

The germline *VHL* testing status of the proband and family members was evaluated. The histories of probands and relatives with confirmed germline *VHL* variants were evaluated for features of VHL syndrome.

### Pedigree evaluation

When available, pedigree was evaluated by assessing affected and genotyped individuals for the co-occurrence of germline *VHL* alleles and VHL phenotype(s). In assessing the strength of possible co-inheritance of the variant with the disease or its *de novo* occurrence, the age of onset of > 60 was considered, given the reported expected high penetrance of deleterious *VHL* [5,^10^,11]. The goal of this step was to identify informative individuals in the pedigree for somatic data assessment. LOD score, linkage disequilibrium and/or determination of segregation with meiosis were not performed.

## Germline variant evaluation

Assessment of the pathogenicity of *VHL* germline sequence variants was performed according to published ACMG/AMP guidelines [1]. This includes a review of relevant and available literature on functional experiments, phenotype presentations of reported cases, *in silico* data, and others as described previously [5]. When available, the observed expression of the *VHL* variants within each family was compared to the reported expected penetrance in literature.

## Tumor signature profile evaluation

The key pattern of tumorigenicity for the *VHL* gene is biallelic inactivation [5,^10^,14]. LOH can be achieved either by germline and somatic contributions (i.e., the first and second hits are germline and somatic respectively) or by a somatic-only contribution (i.e., both the first and second hits are somatic). The second somatic hit is generally a deletion, resulting in LOH, or balanced rearrangement, resulting in copy-neutral LOH. Evaluation of the LOH signature is a key step in this protocol. Given all selected cases are positive for a germline *VHL* variant, careful examination of the tumor signature profile of the *VHL* gene and genomic regions affecting the *VHL* gene is imperative. Therefore, the somatic profiles of patients’ tumors obtained from the OncoPanel test (BWH Pathology, MA, USA) [4,5] was evaluated for single nucleotide variants and indels (SNVs/indels), copy number variants (CNVs), and structural variants (SVs) with a comprehensive multi-tool analysis pipeline. A manual technical review of the pipeline calls was systematically performed in a custom user interface called NGS.Rev to assess the accuracy of alterations and to exclude artifacts.

### Assessment of loss of heterozygosity (LOH)

LOH was used as a signature tumor marker to evaluate the functional consequence of germline variant. The rationale is if the germline *VHL* variant is pathogenic, and any VHL-associated phenotype in the patient is present, the somatic profile is likely positive for an inactivating alteration in the *VHL* gene (i.e., the second hit) consistent with LOH.

In the examination of LOH, careful attention was paid to tumors of these categories: First, tumors exhibiting evidence of somatic biallelic *VHL* inactivation, as they could represent a possible somatic-only contribution to LOH (i.e., both the first and second hits were somatic). An example is clear cell renal cell carcinoma (ccRCC); 96% of these tumors reportedly arise sporadically [10,^14^,15]. Second, tumors with a somatic inactivating alteration in *VHL*, with or without the second somatic hit. An example is hemangioblastoma; 75% of these tumors are reportedly sporadic with or without LOH in the *VHL* gene [16]. In these two categories, the contribution of the germline *VHL* variant could be questionable given that the somatic contribution could fulfil the expected inactivation for the *VHL* gene and/or mechanism of tumorgenicity. Lastly, tumors whose somatic signature was not consistent with the expected LOH of the *VHL* gene were likely not component tumors of the VHL disease (e.g., bladder cancer).

The OncoPanel tumor pipeline does not automatically flag regions with LOH. Zygosity was determined by examining the allele fractions of suspected germline SNVs, including those filtered from the SNV/Indel output obtained from the raw Mutect calls [4]. The variants were plotted according to genomic location, such that areas of LOH dropped out from the 50% allele fraction mark. All true germline SNVs were identified by a separate germline-only pipeline in a commercial laboratory [4,5] (Invitae, CA, USA).

### Assessment of SNV/Indel variants

Somatic SNVs and indels in tumor samples were identified by MuTect and GATK Indelocator (Broad Institute, Cambridge, MA, USA), respectively, as described previously [4,5]. The OncoPanel NGS.Rev interface presents all variants that were not filtered due to presence in a panel of normal samples or those found in the Exome Sequencing Project (ESP) and/or gnomAD databases at >0.1% allele frequency in any sub-population. Any variant filtered by those criteria that was present in the COSMIC database (COSMIC, Wellcome Sanger, London, UK) at least twice was subsequently rescued. The gene, genome coordinates, reference and alternate alleles, coverage, allele fraction, and cDNA and protein change were listed for each variant. An Integrated Genome Viewer (IGV) image link was used to visually evaluate the accuracy of all calls. Somatic OncoPanel was validated with a lower limit of detection of 50Xcoverage and 10% variant allele fraction. Non-filtered artifacts and low coverage and/or allele fraction calls with less than five reads of support were excluded from the analysis.

### Assessment of CNV

Somatic copy number alterations were identified by a custom analysis tool, RobustCNV (DFCI, Boston, MA, USA), as described previously [4,5]. Each baited segment was normalized against the panel of normals, and the Log2 ratios were plotted for visualization in NGS.Rev. Neutral segments had a Log2 ratio of around 0. The overall landscape of a sample's copy number status was assessed in an "all chromosome" view. Each chromosome was then manually reviewed for chromosome-level, arm-level, and/or focal gains or losses. Appropriate calls were entered as low amplifications, high amplifications, one copy deletions, or two copy deletions. In general, low amplifications were called at a Log2 ratio ≥ 0.43 and losses at a Log2 ratio ≤ -0.32.

### Assessment of SV

Somatic chromosomal rearrangements, large indels, and inversions were assessed by BreaKmer, a custom analysis tool (DFCI, Boston, MA, USA) [4,^5^,17] that identifies sequence fragments that do not map to a contiguous region of the reference sequence. BreaKmer-identified sequence fragments were presented in NGS.Rev with the gene(s) involved, genome coordinates, the coverages and numbers of reads supporting the variant, and an IGV snapshot for visual confirmation. Calls whose breakpoints overlapped repetitive regions of the genome were excluded from the analysis. Variants with read support of ≤ 2% (total split and discordant reads/total coverage across breakpoints) were not considered. Variants with greater than 2% support were closely reviewed to confirm the variant was unique to the sample (i.e., not identified in unrelated patients or the normal control).

## Re-evaluation of germline VHL variants using informative somatic genetic signature

In our workflow, individuals positive for germline *VHL* variants who could benefit from somatic tumor evaluation fell in two categories: patients who had tumor(s) that were not component tumors of the VHL syndrome and patients who presented with only one VHL component tumor (Fig. 1).

### Patients with non-VHL tumors

We have previously presented patients with bladder cancer or breast cancer who were positive for heterozygous pathogenic germline *VHL* variants [4,5]. The absence of any VHL phenotype in the personal history of the probands raised questions about the functional role of the germline *VHL* variant that were classified as pathogenic based on ACMG criteria. The tumor genomic signature, i.e., lack of LOH, absence of somatic alterations in the *VHL* genomic regions, and absence of copy-neutral LOH, was consistent with the absence of the VHL phenotype in these patients. This suggested that the germline *VHL* variant did not trigger LOH in these tumors. Altogether, germline *VHL* variants in these patients were likely not deleterious or highly penetrant. It is plausible that the germline *VHL* variants could be affected by genetic modifiers in the genome. Functional studies are required to assess the exact function of variants in each patient.

### Patients with one VHL component tumor

Comprehensive pedigree information, when present, did not support any genotype/phenotype match for VHL. This means other family members were positive for the same *VHL* variants seen in the proband but did not present any tumor or phenotype associated with VHL disease.

In this category, we have presented patients with ccRCC positive for heterozygous pathogenic germline *VHL* variants [4,5]. Tumor profile demonstrated somatic biallelic alterations of VHL (i.e., inactivating somatic SNV in one allele and a one copy deletion in the other allele), consistent with somatic LOH. Given the high frequency of sporadic ccRCC, tumors could be somatic in origin with no or limited germline contribution. Altogether, germline *VHL* variants in these patients are likely not deleterious or highly penetrant.

Another tumor profile showed the presence of one inactivating somatic SNV in one allele in a near-normal diploid genome, with no copy number calls in the second allele. The structural variations profile did not show any rearrangement of *VHL* or evidence of copy-neutral LOH. This profile is consistent with sporadic tumors, e.g., hemangioblastomas, that inactivating mutations in the *VHL* gene is implicated with or without LOH of the *VHL* gene [16]. In these cases, tumors could be somatic in origin with no or limited germline contribution. Germline *VHL* variants in these patients are likely not deleterious or highly penetrant.

If the tumor profile showed mono-allelic loss of *VHL*, it would be plausible that the germline *VHL* variant may in fact be deleterious. The rationale being that the deleterious germline variant was the first “hit” that triggered the one copy loss of *VHL* and the LOH signature. In such cases it can be inferred that both germline and somatic events were involved in LOH. In all cases, functional studies are required to assess the exact function of the variant in each patient.

## Conclusion

The optimized protocol herein leverages tumor-derived somatic data and signature LOH profile as strong supporting evidence in the evaluation of pathogenicity of germline *VHL* variants. Collection of similar cases with detailed tumor derived somatic data can help develop a large database of *VHL* germline variants, that can in turn aid in more accurate clinical assessment and management for patients suspected with VHL syndrome. This integrated evaluation of somatic and germline genetic data can be extended to other cancers, the clinical assessment of which may be improved by refining the interpretation of germline variants.

## Declaration of Competing Interests

The authors declare that they have no known competing financial interests or personal relationships that could have appeared to influence the work reported in this paper.
